# Identification of a conserved 5′-dRP lyase activity in bacterial DNA repair ligase D and its potential role in base excision repair

**DOI:** 10.1093/nar/gkw054

**Published:** 2016-01-29

**Authors:** Ana de Ory, Katja Nagler, Begoña Carrasco, Marina Raguse, Olga Zafra, Ralf Moeller, Miguel de Vega

**Affiliations:** 1 Centro de Biología Molecular ‘Severo Ochoa’ (Consejo Superior de Investigaciones Científicas-Universidad Autónoma de Madrid), Nicolás Cabrera 1, 28049 Madrid, Spain; 2 Radiation Biology Department, German Aerospace Center (DLR), Institute of Aerospace Medicine, Linder Hoehe, D-51147 Cologne, Germany; 3 Centro Nacional de Biotecnología (Consejo Superior de Investigaciones Científicas), Darwin 3, 28049 Madrid, Spain

## Abstract

*Bacillus subtilis* is one of the bacterial members provided with a nonhomologous end joining (NHEJ) system constituted by the DNA-binding Ku homodimer that recruits the ATP-dependent DNA Ligase D (*Bsu*LigD) to the double-stranded DNA breaks (DSBs) ends. *Bsu*LigD has inherent polymerization and ligase activities that allow it to fill the short gaps that can arise after realignment of the broken ends and to seal the resulting nicks, contributing to genome stability during the stationary phase and germination of spores. Here we show that *Bsu*LigD also has an intrinsic 5′-2-deoxyribose-5-phosphate (dRP) lyase activity located at the N-terminal ligase domain that in coordination with the polymerization and ligase activities allows efficient repairing of 2′-deoxyuridine-containing DNA in an *in vitro* reconstituted Base Excision Repair (BER) reaction. The requirement of a polymerization, a dRP removal and a final sealing step in BER, together with the joint participation of *Bsu*LigD with the spore specific AP endonuclease in conferring spore resistance to ultrahigh vacuum desiccation suggest that *Bsu*LigD could actively participate in this pathway. We demonstrate the presence of the dRP lyase activity also in the homolog protein from the distantly related bacterium *Pseudomonas aeruginosa*, allowing us to expand our results to other bacterial LigDs.

## INTRODUCTION

DNA double strand breaks (DSBs) are the most dangerous lesions whose repair is essential for maintenance of genome integrity (1). As bacterial cells often contain multiple partially replicated chromosomes during their vegetative growth, an intact copy of the chromosome is usually available to repair DSBs through the faithful homologous recombination pathway in which the information of the intact duplex is used as template for DNA synthesis across the break (2). However, many bacterial species spend much of their life cycle in stationary phase during which only a single copy of the chromosome is present. In most of these cases the bacterium is also endowed with a two-component nonhomologous end-joining (NHEJ) system (3) that mends DSBs through the direct joining of the DNA ends (3,4). Bacterial NHEJ is composed of the homodimer Ku, homologous to the eukaryotic counterpart (5,6), and the dedicated multifunctional ATP-dependent DNA ligase D (LigD). Extensive characterization of these proteins both *in vitro* and *in vivo* has allowed envisioning how NHEJ operates in bacteria (7–10). Briefly, the NHEJ repair process starts with the recognition and binding of Ku to both sides of the DSB by threading the DNA through its open-ring structure. LigD is further recruited by Ku to mediate the synapsis event required for end-joining. LigD often has a phosphoesterase (PE) activity that heals 3′-ends (11,12); a polymerase activity that fills the gaps that arise after the synapsis; and an intrinsic ATP-dependent ligase activity that finally seals the ends (3,4). Due to the processing of the ends by nucleolytic and/or polymerization activities before final ligation, this pathway is often mutagenic (8,13,14).

Base excision repair (BER) is the most frequently used DNA repair pathway *in vivo* and responsible for the repair of a broad spectrum of non-bulky and non-helix distorting lesions. The increasing number of proteins involved in BER has led to define multiple branches of this repair pathway [see review in (15)]. The general BER process starts with the detection and further removal of the lesion by a specific *N*-glycosylase. The resultant AP site is recognized and processed by AP endonucleases or AP lyases that incise at the 5′ and 3′ sides of the AP site, respectively, requiring further cleaning of the 3′-end by exonucleases and the 5′-dRP terminus by dRPases to leave ligatable 3′-OH and 5′-P termini. A DNA polymerase then closes the gap and a DNA ligase seals the final nick.

*Bacillus subtilis* is a Gram^+^ spore-forming bacterium with a NHEJ system constituted by Ku (*Bsu*Ku) and LigD (*Bsu*LigD) and whose genes are expressed in the developing spore (16). Deletion of those genes sensitizes *B. subtilis* cells to ionizing radiation in the stationary phase (10) and their spores to several DNA-damaging treatments that cause DSBs (16–18). *Bsu*Ku interacts functionally with and stimulates *Bsu*LigD enabling it to generate synaptic intermediates to repair DSBs through the coordinated action of the polymerization and ligase activities (19). Unlike other bacterial LigDs, *Bsu*LigD does not have nuclease activity as it lacks the PE domain. Therefore, in this case other bacterial DNA end-cleaning proteins could heal the 3′-ends. Besides its DNA-binding and *Bsu*LigD recruitment roles, *Bsu*Ku is also provided with an AP/5′-dRP lyase activity that makes this protein able to process ends with near terminal AP sites during the NHEJ pathway (20). The presence of the AP lyase activity in the ortholog from the Gram^−^ bacterium *Pseudomonas aeruginosa* suggests that this activity could be a general feature of bacterial Ku (20), and similar to that of the eukaryotic homolog (21–23).

Although classically repair of AP sites has relied on the recognition and incision of the abasic site by the BER AP endonucleases and further release of the 5′-dRP moiety by the lyase activity of a specialized DNA polymerase, as the eukaryotic polymerases β (24), ι (25), λ (26) and θ (27), there is an increasing number of proteins provided with a 5′-dRP lyase activity that could participate in protecting cells against AP sites, a fact that could reflect the importance for processing such an abundant and deleterious DNA damage [reviewed in (28)]. Thus, in addition to the DNA repair polymerases mentioned above, the *Escherichia coli* DNA polymerase I has been shown to have a dRP-lyase activity although its biological significance has not been established (29). Proteins involved in the nucleotide excision repair (NER) pathway as UvrA, have also been demonstrated to interact with AP-sites, pointing to a potential role of NER as a back-up pathway of AP-sites repair in bacteria (30). Mammalian glycosylases NEIL-1, -2 and -3 are able to remove 5′-dRP lesions at a similar extent of Pol β, and can substitute for Pol β 5′-dRP lyase in an *in vitro* BER assay (31). The mammalian high mobility group proteins HMGA, which are chromatin architectural factors, efficiently remove 5′-dRP groups, protecting cells from DNA damaging agents that cause AP sites (32). Other proteins involved in regulation of the eukaryotic BER as PARP-1 and -2 also show a 5′-dRP lyase although much weaker than the one of Pol β, which is the main processor of 5′-dRP ends during eukaryotic BER (33,34).

Here we show that *Bsu*LigD, besides its polymerization and ligase activities has an inherent and novel 5′-dRP lyase activity. This enables the protein to efficiently perform the gap-filling, 5′-dRP-release and final sealing on a DNA substrate containing an AP site previously incised by an AP endonuclease. Altogether the results lead us to surmise that *Bsu*LigD with a forespore AP endonuclease could constitute a new branch of the BER pathway to mend AP sites during spore germination.

## MATERIALS AND METHODS

### Proteins and reagents

Unlabeled nucleotides were purchased from GE Healthcare. [α^32^P]-Cordycepin (3′-dATP) and [γ^32^P]-ATP were obtained from Perkin Elmer Life Sciences. Substrates were radiolabeled at the 3′ end with [α^32^P]-Cordycepin and terminal deoxynucleotidyl transferase (TdT) or at the 5′ end with [γ^32^P]-ATP and T4 polynucleotide kinase (T4PNK). TdT, T4PNK, human AP endonuclease I (*h*APE1), *E. coli* Uracil DNA Glycosylase (UDG) and *E. coli* EndoIII, were from New England Biolabs. Thrombin was obtained from Novagen. *Bsu*LigD was purified as described (19).

### Preparation of the DNA substrates

To prepare a blunt DNA with an internal 2′-deoxyuridine, a 34-mer oligonucleotide containing 2′-deoxyuridine at position 16 (oligo 1: 5′-CTGCAGCTGATGCGCUGTACGGATCCCCGGGTAC) was either 3′- or 5′-labeled, as indicated, and annealed to its complementary oligonucleotide (oligo 2: 5′-GTACCCGG GGATCCGTACGGCGCATCAGCTGCAG). A gap-filled BER substrate mimicking the situation prior to 5′-dRP release was prepared by hybridizing a templating oligonucleotide (oligo 3: 5′-CCGTACTGCGCATCAGCTGATCACAGTGAGTAC) to a downstream 3′-labeled oligonucleotide (oligo 4: 5′-P-UAGCTGATGCGCAGTACGG) and either to the upstream oligonucleotide 5 (5′-GTACTCACTGTGATC) (hybrid A) or 6 (5′-GTACTCACTGTGATddC) (hybrid B). The 5′-flapped structures were obtained after hybridization of the templating oligonucleotide 7 (5′-CTGCAGCTGATGCGCGTACTCACTGTGATC) to upstream oligonucleotide 8 (5′-GATCACAGTGAGTAC) and either to the 3′-labeled 34-mer downstream oligonucleotide 9 (5′-GTACCCGGGGATCCGTACUGCGCATCAGCTGCAG), that contains 2′-deoxyuridine at position 19 (hybrid C) or to the 3′-labeled 34-mer downstream oligonucleotide 10 (5′-GTACCCGGGGATCCGTACHGCGCATCAGCTGCAG), that harbors a THF (H) at position 19 (hybrid D). Templating oligonucleotide 3 was hybridized to the downstream 3′-labeled oligonucleotide 11 (5′-CTGUAGCTGATGCGCAGTACGG) and to the upstream oligonucleotide 5 to obtain another 5′-flapped structure (hybrid E). The 3′-labeled oligonucleotide 10 was annealed to its complementary oligonucleotide (oligo 12: 5′-CTGCAGCTGATGCGCAGTACGGATCCCCGGGTAC) to obtain a blunt substrate harboring a THF at position 19. To prepare the nicked molecule (hybrid F), a 28-mer templating oligonucleotide (oligo 13: 5′-ACTGGCCGTCGTTGTACTCACTGTGATC) was hybridized to the 5′-labeled 15-mer downstream oligonucleotide 8 and to a 13-mer upstream oligonucleotide (oligo 14: 5′-pAACGACGGCCAGT).

### *In vitro* reconstitution of single-nucleotide BER

Oligonucleotide 1, 3′ or 5′-radiolabeled was hybridized to oligonucleotide 2 to obtain a 34-mer double- stranded DNA substrate. Reactions (12.5 μl) contained 0.53 nM of the hybrid, 30 mM Hepes, pH 7.5, 4% glycerol (v/v), 27 nM *E. coli* UDG, 5 nM *h*APE1, 0.64 mM MnCl_2_ and the indicated concentration of the corresponding nucleotide. Reactions were initiated by adding 57 nM purified *Bsu*LigD, as indicated. Samples were incubated at 30°C for 30 min. After incubation freshly prepared NaBH_4_ was added to a final concentration of 100 mM, and the reactions were further incubated for additional 20 min on ice. Stabilized (reduced) DNA products were ethanol-precipitated in the presence of 0.2 μg/ml tRNA, resuspended in water and analyzed by 8 M urea-20% PAGE and autoradiography.

### 5′-dRP lyase activity on gap-filled BER intermediates

A concentration of 0.96 nM of the indicated hybrid A (upstream primer DNA with a 3′-dCMP) or B (upstream primer DNA with a 3′-ddCMP) was treated with 27 nM *E. coli* UDG for 15 min at 37°C in the presence of 30 mM Hepes, pH 7.5, 4% glycerol. After incubation the mixture was supplemented with 3.5 nM of EndoIII or 60 nM of the indicated LigD or 228 nM of the *Bsu*LigDom in the absence or presence of 0.64 mM MnCl_2_, as indicated. Samples were incubated at 30°C for 30 min and reactions were processed as described in the single-nucleotide BER assay.

### Steady-state kinetic parameters of the dRP lyase reaction

To quantify the kinetic parameters of the 5′-dRP lyase activity, 5′-dRP release was measured as a function of 5′-dRP site concentration, as described in (35,36). Thus, increasing concentrations (0–2000 nM) of hybrid B (upstream primer DNA with a 3′-ddCMP) were treated extensively with *E. coli* UDG (as described above) to render the 5′-dRP group, and further incubated with 50 nM *Bsu*LigD. After incubation for 20 min at 30°C, reaction products were stabilized by incubation with 100 mM of freshly prepared NaBH_4_ for 20 min on ice. Stabilized (reduced) DNA products were ethanol-precipitated in the presence of 0.2 μg/ml tRNA, resuspended in water and analyzed by 8 M urea-20% PAGE and autoradiography. The *k*_obs_ (min^−1^) was plotted against the DNA concentration. Michaelis–Menten constant *K_m_* and *k*_cat_ were obtained by least-squares nonlinear regression to a rectangular hyperbola using Prism 5 software. The values plotted are the mean of three independent experiments.

### NaBH_4_ trapping assay

The 3′ labeled-1/2 hybrid was treated with 27 nM *E. coli* UDG for 15 min at 37°C in the presence of 30 mM Hepes, pH 7.5, 4% glycerol. After incubation, the mixture was supplemented with 5 nM *h*APE1 and 1 mM MnCl_2_ and incubated at 37°C for 30 min. A concentration of 2.6 nM of the resulting DNA was incubated with 95 nM of purified *Bsu*LigD and 10 μM of CTP during 2.5 min, forming a Schiff base intermediate which is trapped by the addition of 100 mM NaCl or freshly prepared NaBH_4_. When indicated, *Bsu*LigD was pre-incubated with 0.05 U of Thrombin in its reaction buffer for 1 h at 20°C in a total volume of 15 μl. After incubation for 30 min on ice, samples were analyzed by 10% SDS-PAGE followed by Coomassie blue staining and autoradiography of the dried gel. When indicated 4.1 nM of the 3′-labeled hybrid E was used as substrate. The hybrid was treated with 27 nM *E. coli* UDG for 15 min at 37°C in the presence of 30 mM Hepes, pH 7.5, 4% glycerol. 4 nM of the resulting DNA was incubated with either 100 nM of purified *Bsu*LigD or *Pae*LigD or 147 nM of LigDom. Samples were processed as mentioned above.

### AP lyase activity assay on 2′-deoxyuridine or THF containing substrates

A concentration of 0.53 nM of the 2′-deoxyuridine-containing hybrids C or D was treated with 27 nM *E. coli* UDG for 15 min at 37°C in the presence of 30 mM Hepes, pH 7.5, 4% glycerol. After incubation the mixture was supplemented with 3.5 nM of EndoIII, 5 nM *h*APE1 or the indicated increasing concentrations of *Bsu*LigD. Samples were incubated at 30°C for 30 min and reactions were processed as described in the single-nucleotide BER assay.

### Cloning and overexpression of *P. aeruginosa* LigD (*Pae*LigD)

The *P. aeruginosa* gene PA2138 encoding *Pae*LigD was synthesized by the GenScript Corporation and cloned between the NdeI and BamHI of bacterial expression vector pET-16b that allows expression of the recombinant protein fused to a N-terminal (His)_10_-tag followed by a thrombin target. *E. coli* BL21(DE3) cells were transformed with the recombinant expression plasmid pET-16*Pae*LigD and grown in LB medium at 37°C in the presence of ampicillin until the A_600_ reached 0.6. Expression of the His-tagged *Pae*LigD protein was induced with 0.5 mM IPTG and further incubation for 20 h at 15°C, as described (37). Cells were thawed and ground with alumina at 4°C. The slurry was resuspended in Buffer A (50 mM Tris-HCl, pH 7.5, 0.7 M NaCl, 7 mM β-mercaptoethanol, 5% glycerol) and centrifuged for 5 min at 6506 x *g*, at 4°C to remove alumina and intact cells. The recombinant *Pae*LigD protein was soluble under these conditions, since it remained in the supernatant after a new centrifugation for 20 min at 234306 x *g*, to separate insoluble proteins from the soluble extract. The soluble extracts were loaded onto a Ni-NTA column (QIAgen) pre-equilibrated with Buffer A (0.7 M NaCl, 4 mM imidazole). The bound protein was eluted with 200 mM imidazole in Buffer A (0.7 M NaCl) and further diluted with Buffer A (1 mM EDTA) without NaCl to get a final 0.3 M NaCl. The sample was applied to a phosphocellulose column preequilibrated with Buffer A (0.3 M NaCl, 1 mM EDTA). The bound protein was eluted with Buffer A (0.4 M NaCl, 1 mM EDTA). The purified protein was finally dialyzed against a buffer containing 0.25 M NaCl and 50% glycerol and stored at −20°C.

### Overexpression of *Bsu*LigD Ligase domain (LigDom)

The recombinant expression plasmid pET28-*Bsu*LigD (19) was used as template to introduce a stop codon at position 320 with the QuikChange site-directed mutagenesis kit provided by Stratagene resulting in plasmid pET28-LigDom**.­­** Cells, previously transformed with plasmid pET28-LigDom, were grown overnight in LB medium at 37°C in the presence of kanamycin. Cells were diluted into the same media and incubated at 30°C until the A_600_ reached 0.6. Then, IPTG (Sigma) was added to a final concentration of 0.5 mM and incubation was continued for 2 h at 30°C. Cells were thawed and ground with alumina at 4°C. The slurry was resuspended in Buffer A (50 mM Tris-HCl, pH 7.5, 0.5 M NaCl, 7 mM β-mercaptoethanol, 5% glycerol) and centrifuged for 5 min at 6506 x *g*, at 4°C to remove alumina and intact cells. The recombinant LigDom was soluble under these conditions, since it remained in the supernatant after a new centrifugation for 20 min at 234306 x *g*, to separate insoluble proteins from the soluble extract. The soluble extracts were diluted with Buffer A without salt to a final 0.25 M NaCl concentration and further loaded onto a Ni-NTA column (QIAgen) pre-equilibrated with Buffer A (0.25 M NaCl, 5 mM imidazole). The bound protein was eluted with 200 mM imidazole in Buffer A (0.25 M NaCl) and further dialyzed against Buffer A (0.3 M NaCl, 50% glycerol, 1 mM EDTA, 0.05% Tween) and stored at −20°C.

### Ligation assay to a 5′-dRP end

3′-labeled hybrid 10/12 was treated with *h*APE1 for 30 min at 37°C in the presence of 30 mM Hepes, pH 7.5, 4% glycerol and 1mM MnCl_2_. The resulting DNA was column purified and 0.53 nM of the nicked substrate was further treated either with *Bsu*LigD or T4 DNA ligase in the presence of 30 mM Hepes, pH 7.5, 4%glycerol. Different concentrations of MnCl_2_ were assayed, in the absence or presence of 0.1 mM ATP. Samples were incubated at 30° C for 30 min and reactions were stopped by adding EDTA up to 10 mM and analyzed by 8M urea-20% PAGE and autoradiography.

### Site-Directed mutagenesis of *Bsu*LigD

*Bsu*LigD mutants K24A, K189A, K206A, K208A and E184A were made by using the QuickChange site-directed mutagenesis kit (Agilent Technologies). Plasmid pET28a-*Bsu*LigD containing the *Bsu*LigD gene was used as template for the reaction (19). The presence of the mutation and the absence of additional ones were determined by sequencing the entire gene. *Bsu*LigD mutants were expressed in *E. coli* SoluBL21^TM^ cells (Genlantis) and further purified as described for the wild-type *Bsu*LigD (19).

### Construction of *B. subtilis* strains expressing the *Bsu*LigD E184A mutant

*Bsu*LigD gene (*ykoU*) containing the mutation E184A was amplified from plasmid pET-*Bsu*LigD-E184A (see above) with a 5′ primer containing an XmaI site and a 3′ primer containing an EcoRV and XbaI sites. The amplified fragment was cloned into the XmaI-XbaI sites of a pUC18 plasmid. *ykoT* gene, placed downstream of *ykoU* was amplified from the *B. subtilis* chromosome with a 5′ primer containing an XbaI and a SacII restriction sites, and a 3′ primer with a PstI site. This gene was cloned into the above plasmid. The pUB110 derived neomicin resistant (Neo^R^) gene *neo* (38) was amplified with a 5′ primer containing an EcoRV and a 3′ primer with a SacI site. The EcoRV-SacII digested *neo* gene was cloned between the *ykoU* (or *ykoUE184A*) and *ykoT* genes in the above pUC18 plasmid. Plasmid-borne *ykoU neo ykoT* or *ykoUE184A neo ykoT* operon was used to transform *B. subtilis* (strain BG214) competent cells, as previously described (38). Neo^R^ transformants were sequenced to select those with the chromosomal-encoded *neo* gene between wild type (wt) *ykoU* and *ykoT* genes (strain BC1000) or between *ykoUE184A* and *ykoT* genes (strain BC1001) (Supplementary Table S1). GP1502 DNA was used to transform BC1000 strain to render the BC1002 strain. Plasmid-borne *ykoUE184A neo ykoT* operon was used to transform the *B. subtilis* BC1002 strain (*Δnfo*) to get strain BC1003 (Supplementary Table S1).

### Bacterial strains and spore preparation

All bacterial strains used in this study are derivatives of 168 strains and are listed in Supplementary Table S1. Spores were obtained by cultivation under vigorous aeration in double-strength liquid Schaeffer sporulation medium (39), and spores were purified and stored as described previously (18,40,41). When appropriate, chloramphenicol (5 μg/ml), kanamycin (10 μg/ml), or erythromycin (2 μg/ml) was added to the medium. Spore preparations consisted of single spores with no detectable clumps and were free (99%) of growing cells, germinated spores and cell debris, as seen with a phase-contrast microscope (18,40,41). The purified spores were resuspended in 5 ml of distilled water and stored until final usage at 4°C.

### Assaying spore resistance to extreme dryness [ultrahigh vacuum (UHV)]

Spore samples consisted of air-dried spore monolayers immobilized on 7-mm in diameter stainless steel discs and were exposed 7 days to UHV produced by an ion-getter pumping system (400l/s; Varian SpA, Torino, Italy) reaching a final pressure of 3 × 10^−6^ Pa (18,41,42). The spores immobilized on quartz discs were recovered by 10% aqueous polyvinyl alcohol solution as described previously (18,42). The appropriate dilutions of treated and untreated spore samples were plated on NB agar plates in order to count CFUs as a measure of spore survival. The CFUs of untreated spore samples were represented as 100% survival. The UHV experiment was performed in triplicate. The CFUs of UHV-treated spores were divided with the average CFU-value of untreated spore samples in order to obtain the survival after UHV. The data presented are expressed as average values with standard deviations. The percentage of survivals of treated spores was compared statistically using Student's t-test and differences with *P*-values of ≤0.05 were considered statistically significant (18,41,42).

## RESULTS

### *Bsu*LigD removes 5′-dRP groups

**Figure 1.**
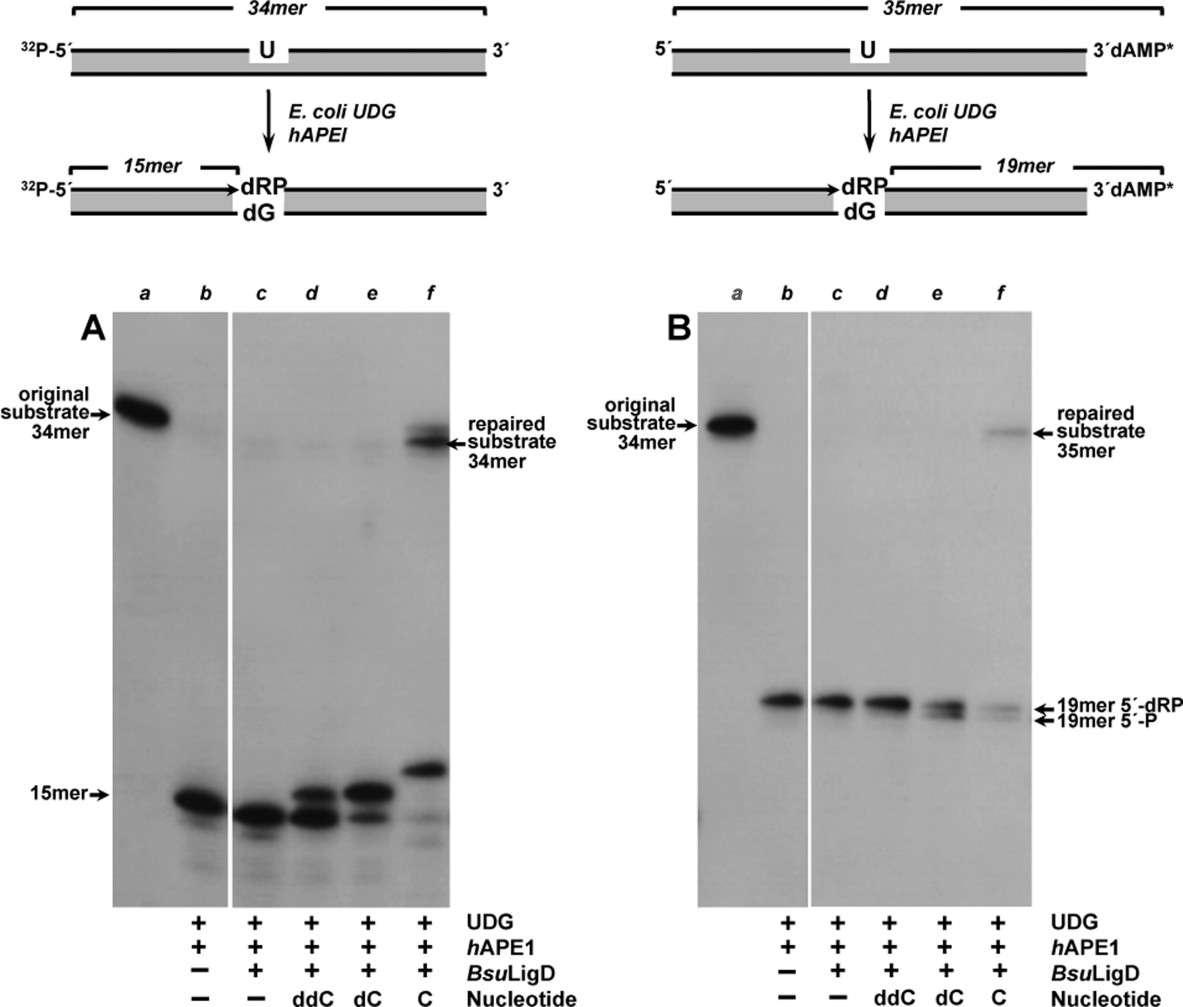
*Bsu*LigD performs complete repair of a BER substrate. Top: schematic representation of the formation of a BER substrate indicating the lengths of the original substrate (^32^P-5′-labeled in left panel or α ^32^P-cordycepin-3′ labeled in right panel) and products after incubation with *E. coli* UDG and *h*APE1. Bottom: autodiagrams illustrating the ability of *Bsu*LigD to repair a BER intermediate. Experiments were performed as described in Materials and Methods. When indicated reactions were incubated in the presence of 57 nM *Bsu*LigD and either 300 μM ddCTP, 10 μM dCTP or 10 μM CTP. After incubation for 30 min at 30°C, samples were analyzed by 8 M urea-20% PAGE and autoradiography. Position of products is indicated. The figure is a composite image made from different parts of the same experiment.

Previous studies showed the ability of the *Bsu*LigD to accommodate to preformed short gaps 1–2 nt long achieving their efficient filling mediated by specific recognition of the 5′-P group at the distal margin of the gap and further sealing of the resultant nick (19). *B. subtilis* AP endonucleases have been reported to be required to repair the AP sites that accumulate during spore dormancy (43–45). This fact suggests that BER should be active during spore germination and outgrowth, and consequently the 1-nt gaps resulting from the action of the AP endonucleases on the abasic sites should be filled by a polymerization activity to allow further sealing of the break. The expression of *Bsu*LigD in the forespore (16) prompted us to gauge the competence of the enzyme to resume gap-filling in BER intermediates where the gap is flanked by a 3′-OH and a 5′-dRP group. To this end, a double-stranded oligonucleotide with a dUMP at position 16 of the ^32^P-5′-labeled strand (see left panel in top of Figure 1, lane *a* in Figure 1A) was treated with *E. coli* UDG to render an AP site. Further incubation with *h*APE1 released a nicked molecule with a 5′-dRP end (opposite to dGMP in the template strand, lane *b* in Figure 1A). As observed, *Bsu*LigD catalyzed efficient template directed addition of both dCMP (lane *e*) and CMP (lane *f*), extending the 75% and 90% of the primer molecules, respectively, discriminating against ddCMP insertion (lane *d*; 29% of the primer molecules extended) as here the formation of the network of direct and water-mediated contacts between the protein and the ribose O2′ and O3′ is precluded (46). Intriguingly, besides the expected +1 (16-mer) elongation product, the enzyme gave rise to a 34-mer product with CTP (corresponding to the 33% of the primer molecules). Direct ligation of the 3′-OH and the 5′-dRP ends can be ruled out as no ligation products were detected in the absence of nucleotides (lane *c*), being tempting to speculate that *Bsu*LigD could remove the 5′-dRP moiety and seal the resulting 5′-P with the 3′-OH group of the elongated primer strand. To test this hypothesis the 3′-end of the U-containing strand was labeled (see right panel in top of Figure 1, lane *a* in Figure 1B). The 5′-dRP end that resulted after treatment with *E. coli* UDG and *h*APE1 remained stable throughout the assay (Figure 1B, lane *b*). As shown, once the gap is filled after insertion of either the deoxy- (lane *e*) or the ribonucleotide (lane *f*), *Bsu*LigD removes 47% and the 68%, respectively, of the 5′-dRP groups as detected by the size reduction of the labeled substrate (19-mer 5′-P), in good agreement with the presence of a dRPase activity in the enzyme. In addition, filling with CTP allowed final ligation of the nick (49% of the 19-mer 5′-P molecules) to yield a repaired 35-mer long molecule (the 3′-labeling excludes that the 35-mer product is the outcome of the complete replication of the template by *Bsu*LigD), reflecting a strong propensity of the enzyme for sealing nicks with a monoribonucleotide on the 3′ end of the break, a functional signature of bacterial NHEJ ligases that distinguishes them from the other polynucleotide ligases (47,48). In this sense, it has been speculated that bacterial NHEJ ligases could be unable to distort the DNA 3′-OH terminus into the RNA-like A conformation observed in other ATP-dependent DNA ligases that do not discriminate between DNA and RNA in the 3′-OH strand (47). Such a distortion would not be required with a 3′-monoribonucleotide, facilitating productive ligation by bacterial LigDs (48).

Altogether the results imply that *Bsu*LigD fills the gap restoring the original (repaired) nucleotide, disclosing a new activity of the protein, the ability to release the dangling 5′-dRP group to generate a canonical and ligatable nick with 3′-OH and 5′-P ends, further sealed by the inherent ligase activity of the enzyme. It is noteworthy that unlike other polymerases involved in gap-filling and 5′-dRP release during BER as eukaryotic polymerases β (24), ι (25), λ (26) and θ (27), *Bsu*LigD is not able to act on the dRP-moiety directly on this substrate (see lane *c* in Figure 1B). The prior filling step requirement would indicate that the optimal substrate for this activity requires the upstream 3′ end to be placed adjacent to the last phosphodiester bond of the downstream strand. In agreement with this hypothesis, the negligible 5′-dRP release observed in the presence of ddCTP (<9%; Figure 1B, lane *d*) would be due to the low primer extension activity observed with this nucleotide (Figure 1A, lane *d*).

### Excision of 5′-dRP groups by *Bsu*LigD proceeds through a β-elimination mechanism

**Figure 2.**
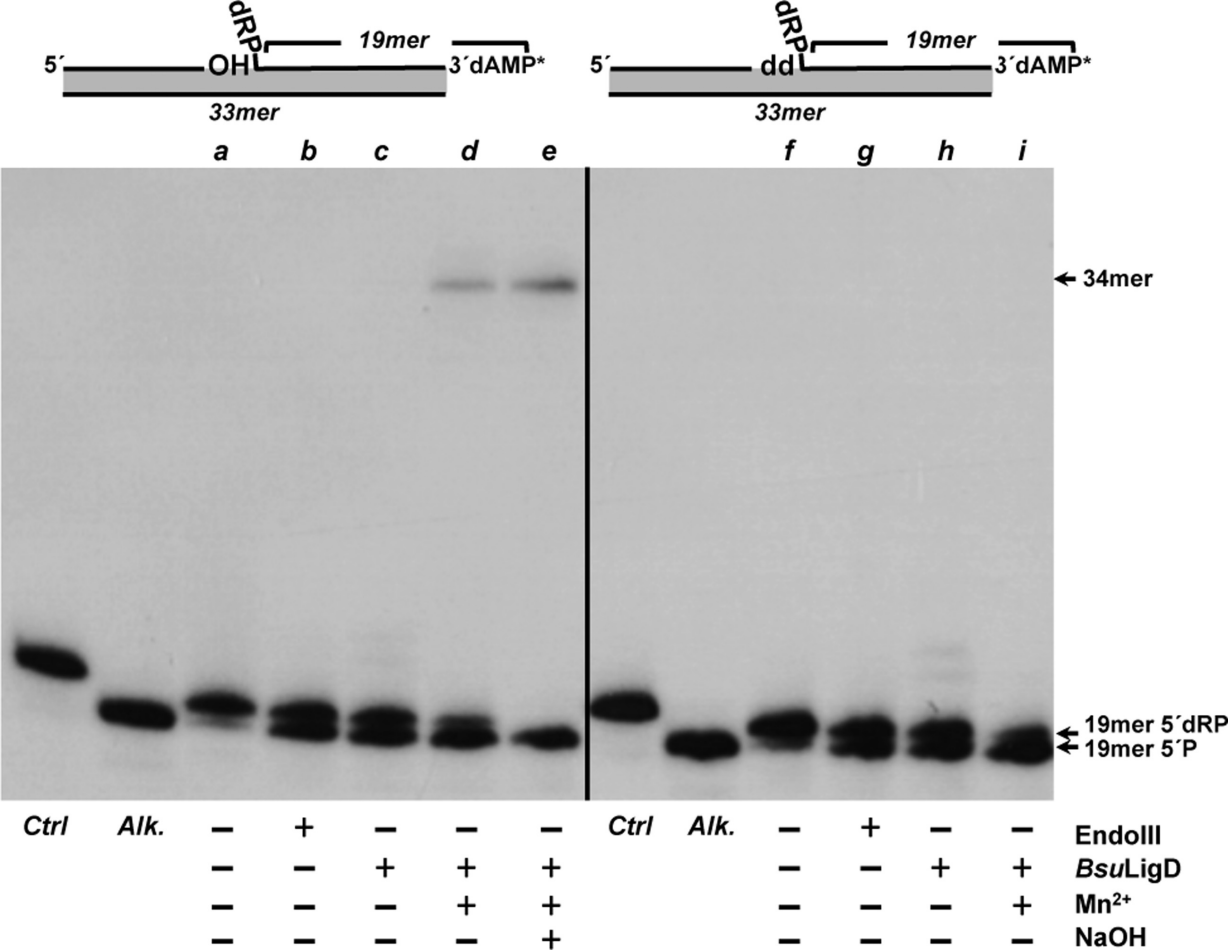
*Bsu*LigD performs non-metal-dependent release of the 5′-dRP moiety. Top: schematic representation of the substrates used in the assay and corresponding to a filled gap with a dangling 5′-dRP group in the downstream strand and either a 3′-OH (left) or dideoxy (right) terminus. Bottom: autodiagrams showing the release of the 5′-dRP group by *Bsu*LigD. Reactions were performed as described in Materials and Methods in the presence of either 3.5 nM EndoIII (lanes *b* and *g*) or 57 nM *Bsu*LigD (lanes *c, d, e, h* and *i*). After incubation during 30 min at 30°C, samples were analyzed by 8 M urea-20% PAGE and autoradiography. Position of products is indicated. Alk, alkaline hydrolysis of the 5′-dRP moiety. Lanes *a* and *f*, original substrate; lanes *c* and *h*, reactions performed in the absence of metal ions; lanes *d* and *i*, reactions performed in the presence of 0.64 mM MnCl_2_; lane *e*, reaction carried out in the presence of 0.64 mM MnCl_2_ and further incubation with alkali. Ctrl lane corresponds to a control of the initial DNA before starting the reaction. The figure is a composite image made from different parts of the same experiment.

In the above assays the requirement of Mn^2+^ ions for the gap-filling step prevented the analysis of the metal dependency of the 5′-dRP release by *Bsu*LigD. Therefore, similar experiments were conducted using a DNA hybrid as substrate mimicking the situation previous to the dRP release, with the 3′-OH end of the upstream strand adjacent to the last phosphodiester bond between the penultimate 5′ nucleotide and the terminal 5′-dRP group of the downstream strand (see scheme at the top of Figure 2). Under these conditions, the absence of divalent cations did not impede the release of the 5′-dRP group by *Bsu*LigD (Figure 2, lane *c*), pointing to a metal independent dRP lyase activity. Although unnecessary, the addition of Mn^2+^ to the reaction improved the dRPase activity of *Bsu*LigD (Figure 2, lane *d*). Maybe the presence of this metal ion (the preferred cation for both the polymerization and ligase activities of *Bsu*LigD (19)) assists the stable/proper binding of the protein to the DNA substrate, as described for the 5′-dRP lyase activity of Pol β (24,36). As shown, further addition of alkali did not hydrolyze the 34-mer product, supporting the notion that the repaired DNA was not the result of a direct ligation of the upstream strand to the 5′-dRP group (Figure 2, lane *e*). Similar results were obtained with a substrate bearing a ddNMP at the 3′ end of the upstream strand (see Figure 2, right panel). As expected, in this case no ligation products were observable. These results indicate that the dRP-release activity is not the result of an in-line attack of the last phosphodiester bond by the 3′-OH group that could mimic the mode of action of DNA ligases (47).

**Figure 3.**
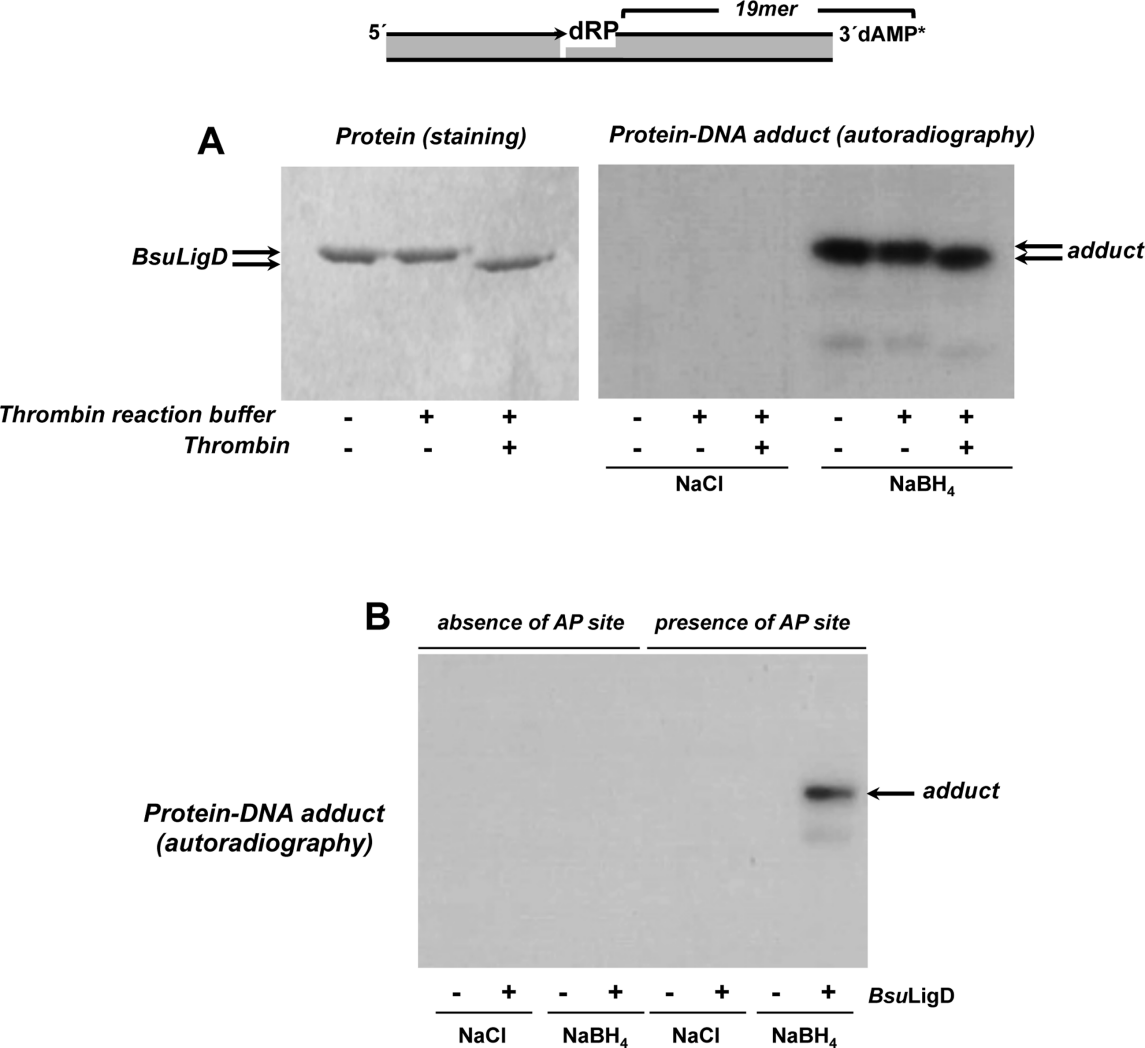
Formation of *Bsu*LigD-DNA adducts. (**A**) Dependence of *Bsu*LigD-DNA cross-link on NaBH_4_. Reactions were performed as described in Materials and Methods, incubating 95 nM *Bsu*LigD with 2.6 nM of the 3′ [α^32^P]3′-dAMP labeled DNA substrate depicted on top of the figure, in the presence of 10 μM CTP, 0.64 mM MnCl_2_ and either 100 mM NaBH_4_ or NaCl (as indicated). Left panel: Coomassie blue staining after SDS–PAGE of purified *Bsu*LigD. Right panel: autoradiography of corresponding protein-DNA adducts after the SDS–PAGE separation shown in left panel. When indicated, protein was previously incubated with 0.05 U of thrombin at 20°C for 60 min. (**B**) Adduct formation is dependent on the presence of an abasic site. Reactions were performed as in described in (A) but using as substrate 3.6 nM of the 3′ [α^32^P]3′-dAMP labeled oligonucleotide without removing the uracil (*absence of AP site*) or after treatment with *E. coli* UDG (*presence of AP site*), in the presence of either 100 mM NaBH_4_ or NaCl (as indicated). Autoradiography of corresponding protein-DNA adduct after the SDS–PAGE separation is shown.

5′-dRP release by DNA polymerases β, ι, λ, θ and γ proceeds through β-elimination, a mechanism that involves generation of a Schiff-base intermediate and that allowed categorizing the activity as a 5′-dRP lyase (24–27,35). To elucidate whether this was also the case with *Bsu*LigD, we took advantage of the ability of NaBH_4_ to reduce a Schiff-base intermediate to form a covalent protein-DNA complex. Therefore, if the mechanism of catalysis of *Bsu*LigD involves a Schiff-base intermediate, addition of NaBH_4_ to the gap-filling reaction described above should permit trapping of a DNA-protein complex that would be detected by autoradiography after separation by SDS-PAGE. As shown in Figure 3A, *Bsu*LigD forms a stable adduct with the 3′ labeled 5′-dRP-containing 19-mer strand that was dependent on both, addition of NaBH_4_ and presence of an AP site in the DNA (Figure 3B). These results indicate that the 5′-dRP removal activity of *Bsu*LigD proceeds through β-elimination. Removal of the fused N-terminal His-tag from *Bsu*LigD after incubation with thrombin gave rise to DNA-*Bsu*LigD adducts whose faster migration paralleled the electrophoretical pattern of the purified protein, indicating that the 5′-dRP lyase activity is intrinsic to *Bsu*LigD (Figure 3A) and ruling out the presence of a contaminant AP lyase from the expression bacteria *E. coli*.

The presence of nonenzymatic AP lyase activity has been described in basic cellular macromolecules such as polyamines or histones and in other basic molecules including tripeptides such as Lys-Trp-Lys and Lys-Tyr-Lys (49), although cleavage generally occurs at a very low efficiency (50). To ascertain that the 5′-dRP lyase activity exhibited by LigD was indeed catalytic the activity was assayed under steady-state conditions as described in (35,36) on the above DNA hybrid. The apparent *K_m_* for this DNA and the *k_cat_* were 2 ± 0.65 μM and 0.88 ± 0.17 min^−1^, respectively (see Supplementary Figure S1). Therefore, *Bsu*LigD *k_cat_* is 5-fold lower than that of Pol β assayed on preincised AP-DNA (4.5 min^−1^) (36), but still 3-fold higher than the *k_cat_* of the 5′-dRP lyase activity of Pol λ (0.26 min^−1^) (35). These results, together with the coupling of the 5′-dRP lyase activity to polymerization and its improvement in the presence of Mn^2+^ ions, lead us to conclude that the 5′-dRP lyase of *Bsu*LigD is catalytic.

**Figure 4.**
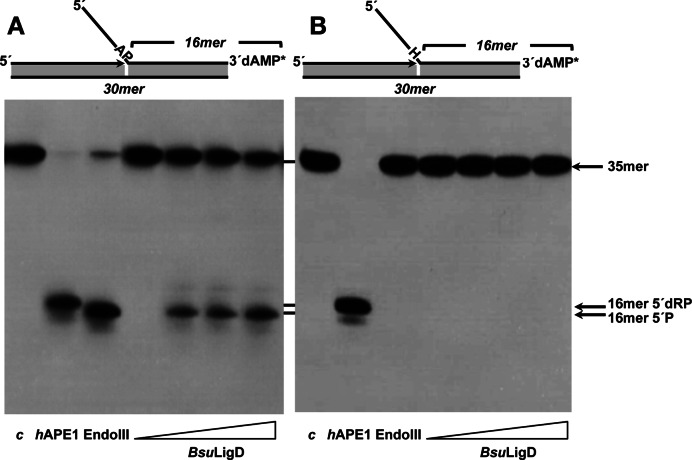
*Bsu*LigD is endowed with an AP lyase activity. (**A**) Analysis of the capacity of *BsuL*igD to incise an internal natural abasic site. The [α^32^P]3′-labeled 2′-deoxyuridine-containing substrate was treated with 27 nM *E. coli* UDG (lane *c*), leaving an intact AP site. The resulting AP-containing DNA was incubated in the presence of either 5 nM *h*APE1 that cleaves 5′ to the AP site, 3.5 nM EndoIII that incises 3′ to the AP site, or increasing concentrations of *Bsu*LigD (0, 29, 57 and 114 nM) for 1 h at 30°C, as described in Materials and Methods. After incubation samples were analyzed by 8 M urea-20% PAGE and autoradiography. Position of products is indicated. (**B**) Analysis of the capacity of *Bsu*LigD to incise an internal tetrahydrofuran (H). The 3′ [α^32^P]3′-dAMP labeled oligonucleotide containing the lyase-resistant analogue tetrahydrofuran (H) was incubated in the presence of either *h*APE1, EndoIII or increasing concentrations of *Bsu*LigD as described above. Position corresponding to the products 16-mer 5′-dRP and 16-mer 5′-P is indicated. The figure is a composite image made from different parts of the same experiment.

The capacity of *Bsu*LigD to release a 5′-dRP group led us to evaluate its ability to recognize and incise an internal AP site, as traditionally 5′-dRP lyases have been considered a subset of AP lyases (51). To this end, the flapped DNA structure depicted in left panel of Figure 4 and containing a 2′-deoxyuridine at position 19 of the 35-mer downstream oligonucleotide was used as substrate. This DNA was previously treated with *E. coli* UDG to get a natural AP site. Incubation of this substrate with *h*APE1 rendered a 16-mer product with a 5′-dRP end (Figure 4, left panel) as this enzyme is a metal-dependent AP endonuclease that hydrolyzes the phosphodiester bond 5′ to the AP site [(52) and references therein]. Conversely, *E. coli* EndoIII incised at the 3′ side by its AP lyase activity leaving a product that migrates faster due to the presence of a 5′-P [(52) and references therein]. As shown in Figure 4 (left panel), in the absence of divalent cations incubation of the AP site-containing DNA with increasing amounts of *Bsu*LigD rendered a product with the same electrophoretical mobility to that produced by EndoIII, consistent with a cleavage at the 3′ side to the AP site in a metal-independent manner. In this sense, the presence of the AP cleavage activity after incubating the protein overnight with up to 100 mM EDTA (see Supplementary Figure S2), allows us to rule out metal traces as responsible for such an activity, in agreement with the metal independent 5′dRP lyase activity described above. These results lead us to infer the presence of an intrinsic AP lyase activity in *Bsu*LigD that exerts its reaction through a β-elimination mechanism. In support of this, replacement of the AP site with tetrahydrofuran (THF), a stable AP analog resistant to the β-elimination reaction (24,28) inhibited the *Bsu*LigD activity (Figure 4, right panel).

### The presence of a 5′-dRP lyase activity is conserved in other bacterial LigDs

**Figure 5.**
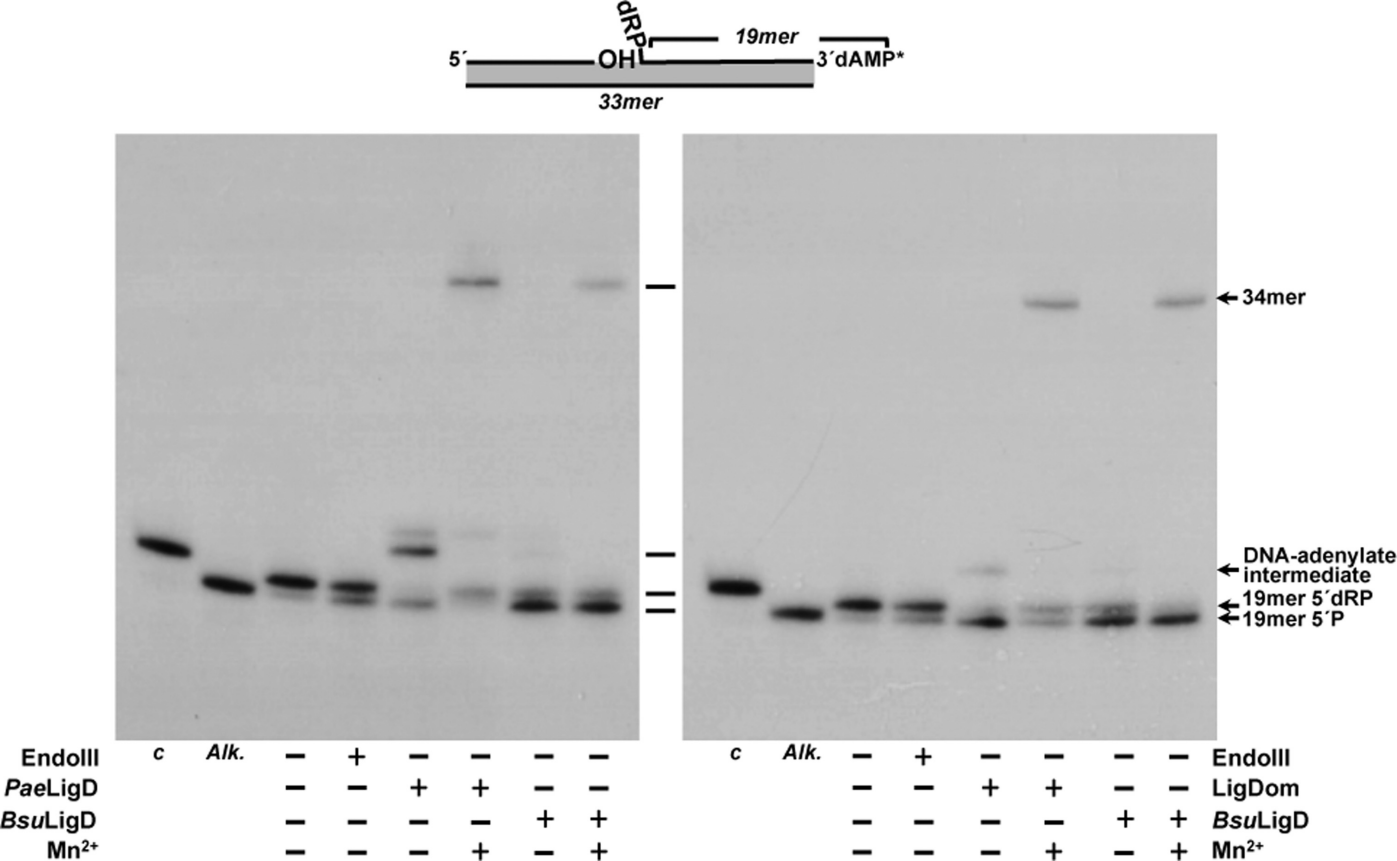
Left: *Pae*LigD is endowed with a 5′-dRP lyase activity. The assay was performed as indicated in Materials and Methods in the presence of either 3.5 nM of EndoIII or 60 nM of the indicated LigD in the absence (−) or presence (+) of 0.64 mM MnCl_2_. After incubation during 30 min at 30°C samples were analyzed by 8 M urea-20% PAGE and autoradiography. Position of products is indicated. *Alk*, alkaline hydrolysis of the 5′-dRP moiety. Right: the 5′-dRP lyase activity of *Bsu*LigD resides in the ligase domain. The assay was performed as in left panel in the presence of 216 nM LigDom. After incubation during 30 min at 30°C samples were analyzed by 8 M urea-20% PAGE and autoradiography. Position of products is indicated. The figure is a composite image made from different parts of the same experiment.

The unforeseen presence of a 5′-dRP lyase activity in *Bsu*LigD led us to analyze whether this activity is specific to the *B. subtilis* protein or, by the contrary if its presence can be extended to other bacterial LigDs. To this end, we purified the 94 kDa *Pseudomonas aeruginosa* LigD (*Pae*LigD; see Materials and Methods) since (i) it has been used as model for bacterial LigDs for years (3,4), (ii) shows a configuration different from *Bsu*LigD because it contains an additional N-terminal PE domain and (iii) it comes from a Gram^−^ bacterium. As shown in Figure 5 (left panel), the purified *Pae*LigD possesses a non metal-dependent 5′-dRP lyase activity since it releases the 5′dRP moiety from the 3′-labeled substrate yielding the 19-mer 5′P product that is adenylated at some extent by the proportion of the AMP-*Pae*LigD complexes coming from the expression bacterium, as described (20,48,53). In the presence of Mn^2+^ ions *Pae*LigD rendered a repaired 34-mer ligation product. As shown in Supplementary Figure S3, purified *Pae*LigD is cross-linked to the DNA after reduction with NaBH_4_. Altogether, the results allow us to widen the presence of a 5′-dRP lyase activity to other bacterial LigDs.

### The 5′-dRP lyase activity of *Bsu*LigD is located at the N-terminal ligase domain

Previous studies on ATP-dependent DNA ligases from bacteriophages T4 and T7 as well as from the human mitochondria showed that those enzymes were endowed with an intrinsic 5′-dRP lyase activity (54,55). As mentioned above, *Bsu*LigD is a bimodular enzyme with an N-terminal ATP-dependent DNA ligase catalytic domain (residues 1–331) linked to a C-terminal polymerase domain (residues 332–611). Thus, it was reasonable to speculate that the *Bsu*LigD 5′-dRP lyase activity could reside at the N-terminal portion of the enzyme. To test this hypothesis, the ligase domain of *Bsu*LigD (LigDom) was cloned and purified (see Materials and Methods). As shown in Figure 5 (right panel), the LigDom released the 5′-dRP group from the substrate in a metal-independent manner, giving rise to a ligation product in the presence of Mn^2+^. As shown in Supplementary Figure S3, LigDom is also cross-linked to the DNA following reduction with NaBH_4_. These results indicate that the catalytic site responsible for *Bsu*LigD AP lyase activity is placed at the LigDom.

In contrast to T4 DNA ligase, *Bsu*LigD is unable to seal the 3′-OH and 5′-dRP ends to regenerate an internal AP site (see Supplementary Figure S4, in this case, the substrate harbors a THF to prevent the β-elimination). It has been predicted that once regenerated, T4 DNA ligase could recognize the internal AP site and exert its AP lyase activity introducing an incision at the 3′ side (54). As a consequence, the resulting 3′-phospho-α,β-unsaturated aldehyde end should be processed by additional nucleolytic activities to regenerate an elongatable 3′-OH group. Therefore, prevention of direct ligation by *Bsu*LigD could represent an advantage as the enzyme is compelled to accomplish previous 5′-dRP release, precluding the need for additional activities.

The formation of a stable protein–DNA substrate adduct between *Bsu*LigD and 5′-dRP-containing DNA in the presence of a reducing agent is consistent with the AP lyase active site lysine residue forming a Schiff base intermediate with the open-ring form of the abasic site. Thus, to determine whether the ligase and the lyase activities use the same active site we have changed into alanine the *Bsu*LigD residues Lys24 (mutant K24A), Lys189 (mutant K189A), Lys206 (mutant K206A) and Lys208 (mutant K208A) as their homologous residues Lys481, Lys618, Lys635 and Lys637 of *Mycobacterium tuberculosis* LigD (*Mtu*LigD) have been shown to form part of the ligation active site (56). In addition, *Bsu*LigD Glu184, the counterpart of the metal ligand Glu613 of *Mtu*LigD, one of the catalytic residues responsible for the ligation activity (56) was also mutated to alanine (mutant E184A). As shown in Supplementary Figure S5, all the mutant derivatives were deficient in the ligation activity, as expected, but retained a 5′-dRP lyase activity similar to that of the wild-type enzyme. These results led us to conclude that both activities are not sharing the same active site.

### Spore resistance after UHV treatment depends on *Bsu*LigD and Nfo

**Figure 6.**
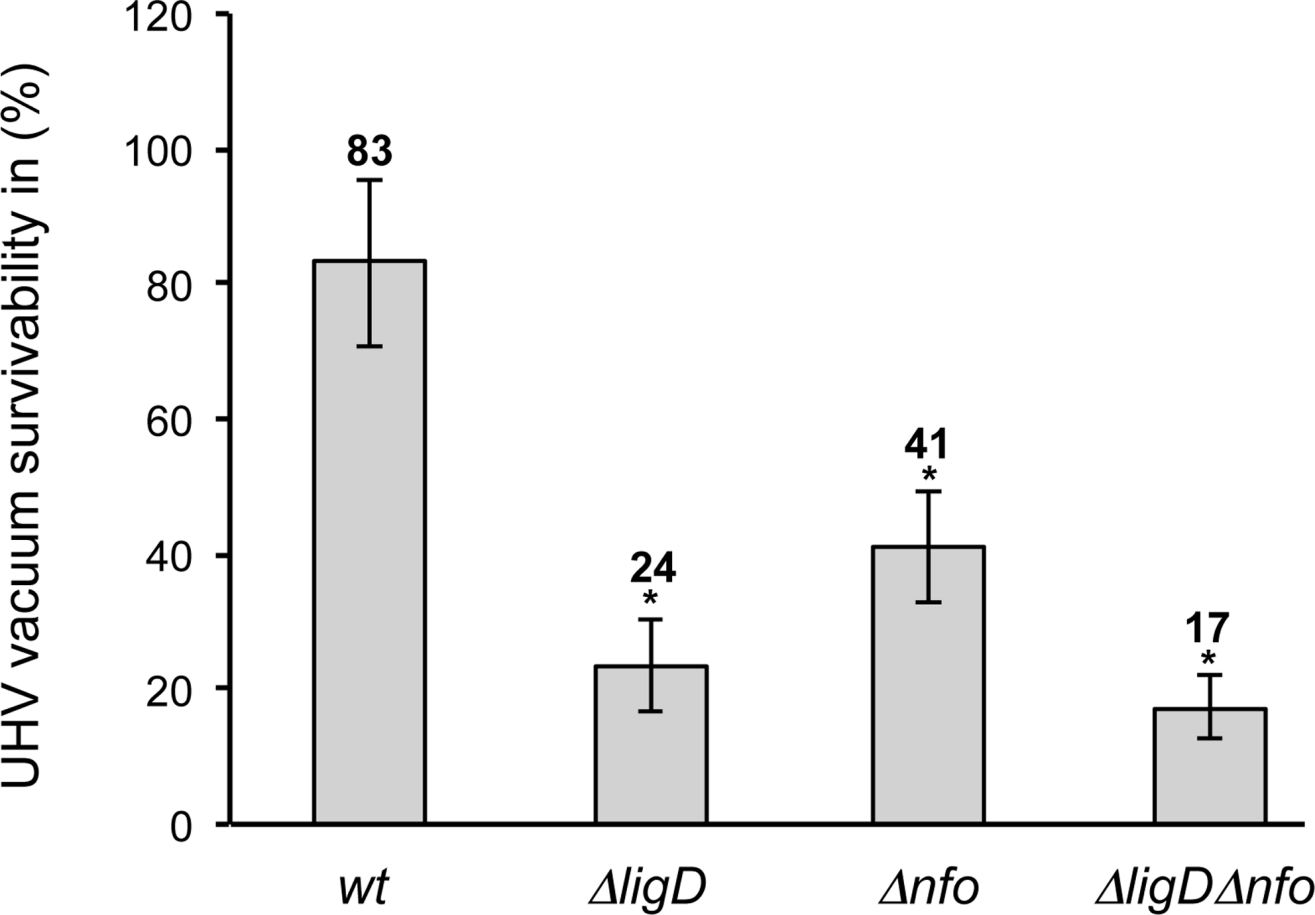
Survival of *B. subtilis* spores deficient in *Bsu*LigD and/or Nfo AP endonuclease. The assay was performed as described in Materials and Methods. The CFUs of UHV-treated spores were divided with the average CFU-value of untreated spore samples in order to obtain the survival after UHV. The data presented are expressed as average values ± SD, *N* = 3. Asterisks indicate UHV survival values that were significantly different (*P*-values ≤ 0.05) from values for wild-type (wt) spores.

Previous studies showed that deletion of *B. subtilis* AP endonucleases sensitized spores to desiccation in agreement with the induction of single-stranded nicks (43). In addition, ultrahigh vacuum (UHV) desiccation also decreased the survivability of the Δ*ligD* mutant spores, which was consistent with the induction also of DSBs in DNA. In order to determine a potential relationship between *Bsu*LigD and the BER pathway, *B. subtilis* mutant spores lacking *Bsu*LigD (Δ*ligD*), the spore-specific AP endonuclease IV Nfo (Δ*nfo*) and Δ*ligD*Δ*nfo* were subjected to UHV desiccation treatment. As shown in Figure 6, Δ*ligD* single mutation caused a 4-fold reduction in spore survival. Similarly, deletion of the spore AP endonuclease Nfo caused a 2-fold increase of the sensitivity of the spores. These results indicate the involvement of both *B. subtilis* proteins in the DNA repair in spores after UHV exposure for 7 days. Interestingly, *B. subtilis* LigD and Nfo do not appear to contribute additively to spore resistance after UHV treatment as Δ*ligD*Δ*nfo* rendered spores with a sensitivity statistically similar to that displayed by the single mutant Δ*ligD*, suggesting a functional interaction between both repair proteins. Similar results were obtained after testing the effects of the ligase-inactivating *Bsu*LigD E184A mutation on the repair efficiency of the UHV induced lesions (see Supplementary Figure S6). Altogether, the results support the presence of a spore specific BER pathway to repair abasic lesions during spore germination, in which *Bsu*LigD plays a pivotal role.

## DISCUSSION

*B. subtilis* spores are continuously exposed to environmental conditions that cause the accumulation of potentially lethal and mutagenic DNA lesions such as the spore photoproduct, strand breaks, cyclobutane pyrimidine dimers, altered bases and AP sites (57). In addition, AP sites can be also generated during spore germination and outgrowth either after removal of a damaged base by a specific glycosylase (58) or after spontaneous breakage of the *N*-glycosidic bond under physiological conditions (59). Therefore, the spore should be provided with the machinery required to recognize and repair those lesions during germination and outgrowth to prevent mutagenesis as well as potential stalling of the replication and transcriptional machineries that could lead to chromosome breakage (52). In this sense, *B. subtilis* gene *nfo*, which encodes for AP endonuclease IV (Nfo), is expressed under the control of the σ^G^ transcription factor late in sporulation (43) and the protein is present in mature spores. In addition, the levels of β-galatosidase from an *exoA-lacZ* translational fusion showed that expression of *exoA* which codes for AP endonuclease ExoA also takes place during sporulation (43), although in this case it remains to be determined whether the protein is present in the dormant spore. The absence of ExoA and/or Nfo in deletion mutant strains of *B. subtilis* sensitized the spores to treatments that damage spore DNA through generation of AP sites and strand breaks, suggesting that BER should be active to repair the lesions during spore germination and outgrowth that have accumulated during spore dormancy (43–45). The action of these AP endonucleases on AP sites renders a gap flanked by 3′-OH and a 5′-dRP ends. Accomplishment of AP site repair would require a polymerization step to close the gap, a 5′-dRPase to render a ligatable 5′-P and a ligase activity to seal the final nick. *B. subtilis ykoU* gene codes for *Bsu*LigD and forms part of a regulon under the control of both, the RNA-polymerase sigma factor σ^G^ and the DNA-binding protein SpoVT, and whose expression is turned on in the forespore (16). We have shown here that *Bsu*LigD could potentially participate in BER since the enzyme (i) efficiently fills a single nucleotide gap on preincised AP-DNA, (ii) removes the 5′-dRP group by an intrinsic lyase activity rendering a nick with ligatable 3′-OH and 5′-P ends, (iii) seals the break and (iv) seems to participate together with the *B. subtilis* spore AP endonuclease Nfo in the repair of DNA lesions induced by UHV desiccation. The ability of *Bsu*LigD to fill the gap prior to dRP-release, as well as its failure to seal 3′-OH and 5′-dRP ends would guarantee the repair of the lesion without loss of sequence information. The absence in our reconstitution assays of accessory factors indicates that the polymerization, dRP lyase and ligation functions of *Bsu*LigD could be necessary and sufficient for ‘short patch’ BER of AP sites during spore germination and outgrowth together with the *B. subtilis* AP endonucleases Nfo and/or ExoA. Therefore, although *a priori* the bacterial *Bsu*LigD complex had been exclusively involved in the repair of DSBs through the NHEJ pathway, the results presented here are suggestive of a potential participation of this protein in bacterial BER as well, a hypothesis that could be extended to the rest of bacteria in the light of the results obtained with the LigD from *P. aeruginosa*, and maybe to the recently reported archaeal NHEJ DNA Lig (60). Altogether, our observations suggest that the role of the ATP-dependent ligase domain is not restricted to the final strand closure, paving the way to future works aimed to decipher the *in vivo* and *in vitro* interplay with other DNA repair proteins of the BER pathway. Interestingly, recent results have implied a Ku-independent role of *Pseudomonas putida* LigD in stationary-phase mutagenesis that led authors to surmise the involvement of LigD in other DNA metabolism-related processes that use translesion synthesis and/or gap-filling on damaged DNA (61). A dual role of NHEJ protein factors has been also documented in eukaryotes where polymerases responsible for NHEJ contribute to a short-patch BER that repairs damage-associated chromosome breaks (62). Recently it has been shown how deletion of the mice Ku70 or Ku80 results in different sensitivities of cells to genotoxicants that provoke DNA lesions as alkylated and oxidized bases and single-strand breaks that are repaired by the BER pathway (63,64).

We have shown that the active site responsible for the AP lyase activity resides in the N-terminal ligase domain. The presence of a 5′-dRP lyase activity was previously described in the ATP-dependent DNA ligases from bacteriophages T4 and T7 (54) as well as from the human mitochondria (55). Evolutionary studies suggest that all ATP-dependent DNA ligases descend from a common ancestor and show six conserved sequence motifs (I, III, IIIa, IV, V-VI) that define a family of related nucleotidyltransferases [reviewed in (65)]. The occurrence of a 5′-dRP lyase activity in the ATP-dependent ligase domain of bacterial LigD led us to venture the presence of an AP lyase activity as a general feature of at least the ATP-dependent DNA ligases.

## Supplementary Material

SUPPLEMENTARY DATA
