# Supplementary material for: Identification of a conserved 5′-dRP lyase activity in bacterial DNA repair ligase D and its potential role in base excision repair
Source: Nucleic Acids Res. 2016 Jan 29;44(4):1833–44. doi: 10.1093/nar/gkw054 (PMC4770248; doi:10.1093/nar/gkw054)
Supplement: SUPPLEMENTARY DATA [file supp_44_4_1833__index.html]

Identification of a conserved 5′-dRP lyase activity in bacterial DNA repair ligase D and its potential role in base excision repair — SUPPLEMENTARY DATA 

# Identification of a conserved 5′-dRP lyase activity in bacterial DNA repair ligase D and its potential role in base excision repair

## SUPPLEMENTARY DATA

- SUPPLEMENTARY DATA
