# Supplementary material for: Identification of a conserved 5′-dRP lyase activity in bacterial DNA repair ligase D and its potential role in base excision repair
Source: Nucleic Acids Res. 2016 Jan 29;44(4):1833–44. doi: 10.1093/nar/gkw054 (PMC4770248; doi:10.1093/nar/gkw054)
Supplement: SUPPLEMENTARY DATA [file supp_gkw054_nar-02220-d-2015-File008.pdf]

Supplementary Information

Identification of a Conserved 5´-dRP Lyase Activity in Bacterial DNA Repair Ligase D and Its Potential Role in Base Excision Repair

Ana de Ory[1], Katja Nagler[2], Begoña Carrasco[3], Marina Raguse[2], Olga Zafra[1], Ralf Moeller[2,]* and Miguel de Vega[1,]*

[1]Centro de Biología Molecular “Severo Ochoa” (Consejo Superior de Investigaciones Científicas-Universidad Autónoma de Madrid), Nicolás Cabrera 1, 28049 Madrid, Spain.
[2] Radiation Biology Department, German Aerospace Center (DLR), Institute of Aerospace Medicine, Linder Hoehe, D-51147 Cologne, Germany.
[3]Centro Nacional de Biotecnología (Consejo Superior de Investigaciones Científicas), Darwin 3, 28049 Madrid, Spain.
*To whom correspondence should be addressed. Tel: +34 911964717; Fax: +34 911964420; Email: mdevega@cbm.csic.es. Correspondence may also be addressed to Tel: +49 2203 601 3145; Fax: +49 2203 61790; E-mail: ralf.moeller@dlr.de

Table S1. Bacterial strains used in this study

Strain	Gene/mechanism	Genotype	Source/reference
168	wt	*trpC2*	Laboratory strain
BFS184	LigD [NHEJ]	+ *ykoU*:*erm*[a]	(1)
GP1502	Nfo [EndoIV]	+ *Δnfo*:*cat*[b]	(2)
GP1502L	LigD Nfo	+ *ykoU*:erm Δ*nfo*:cat[c]	GP1502 DNA transform BFS1845
BG214	wt	*trpCE metA*5 *amyE1 ytsJ*1 *rsbV*37 *xre*1 *xkd*A1 *att*^{SPß} *att*^{ICEBs1}	Laboratory strain
BC1000	wt	+ *neo*[d]	This work
BC1001	LigDE184A	+ *ykoU*E184A *neo*[d]	This work
BC1002	Nfo	+ *Δnfo*:*cat*[b] *neo*[d]	This work
BC1003	LigDE184A Nfo	+ *ykoU*E184A *neo*[d] *Δnfo*:*cat*[b]	This work

[a]Resistance to erythromycin (Erm^{R}, 2 μg/ml), [b]Resistant to chloramphenicol (Cam^{R}, 5 μg/ml), [c]Erm^{R} (2 μg/ml) and Cam^{R} (5 μg/ml), [d] Resistant to neomycin^{R} (Neo^{R}, 5 μg/ml).

References

1. Weller GR, Kysela B, Roy R, Tonkin LM, Scanlan E, *et al.* (2002) Identification of a DNA nonhomologous end-joining complex in bacteria. *Science* **297**(5587):1686-1689.
2. Gunka K, Tholen S, Gerwig J, Herzberg C, Stulke J, *et al.* (2012) A high-frequency mutation in Bacillus subtilis: requirements for the decryptification of the gudB glutamate dehydrogenase gene. *J Bacteriol* 194(5):1036-1044.

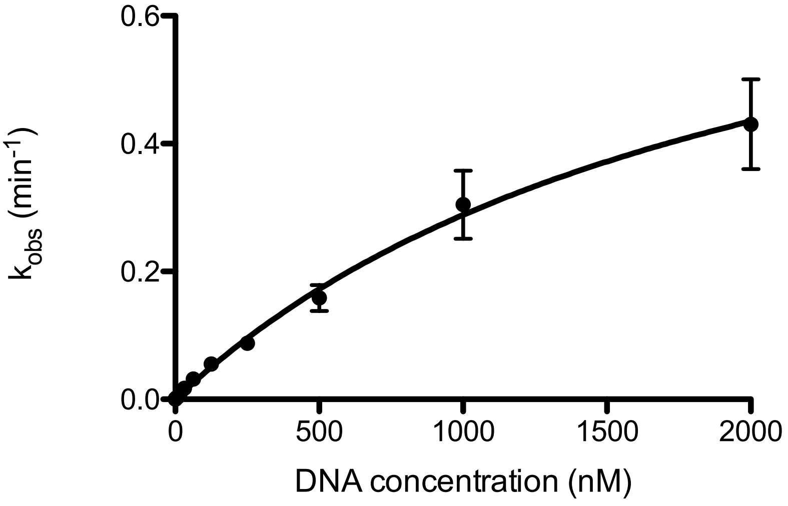

Supplementary Figure S1. Steady-state kinetic analysis of the 5´-dRP lyase activity of *Bsu*LigD. The release of dRP from a 3´ labeled 5´-dRP-containing DNA substrate was examined as a function of the indicated substrate concentration as described in Materials and Methods. Reactions contained 50 nM *Bsu*LigD and were incubated at 30 ºC for 20 min. Apparent activities were fitted to the Michaelis equation by least-squares nonlinear regression. The values plotted are the mean of three independent experiments.

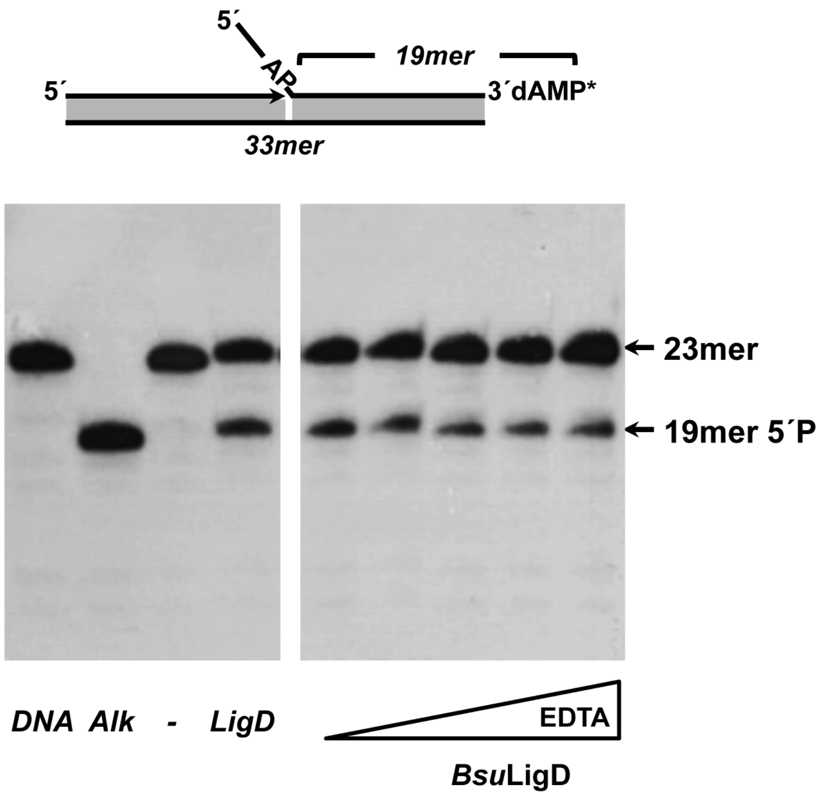

Supplementary Figure S2. *Bsu*LigD AP-lyase activity in the presence of EDTA. 0.83 nM of the [^{32}P]3′-labeled uracil-containing hybrid E depicted on top of the figure was treated with *E. coli* UDG, leaving an intact AP site (*DNA*). 57 nM *Bsu*LigD was preincubated with increasing concentrations of EDTA (0, 12.5, 25, 50 and 100 mM). After overnight incubation at 4ºC, both the EDTA-treated *Bsu*LigD and 57 nM of fresh protein were incubated with the DNA substrate during 30 min or 30ºC, as described in Materials and Methods. After incubation samples were analyzed by 8 M urea-20% PAGE and autoradiography. Position of products is indicated. *Alk,* alkaline hydrolysis of the AP site.

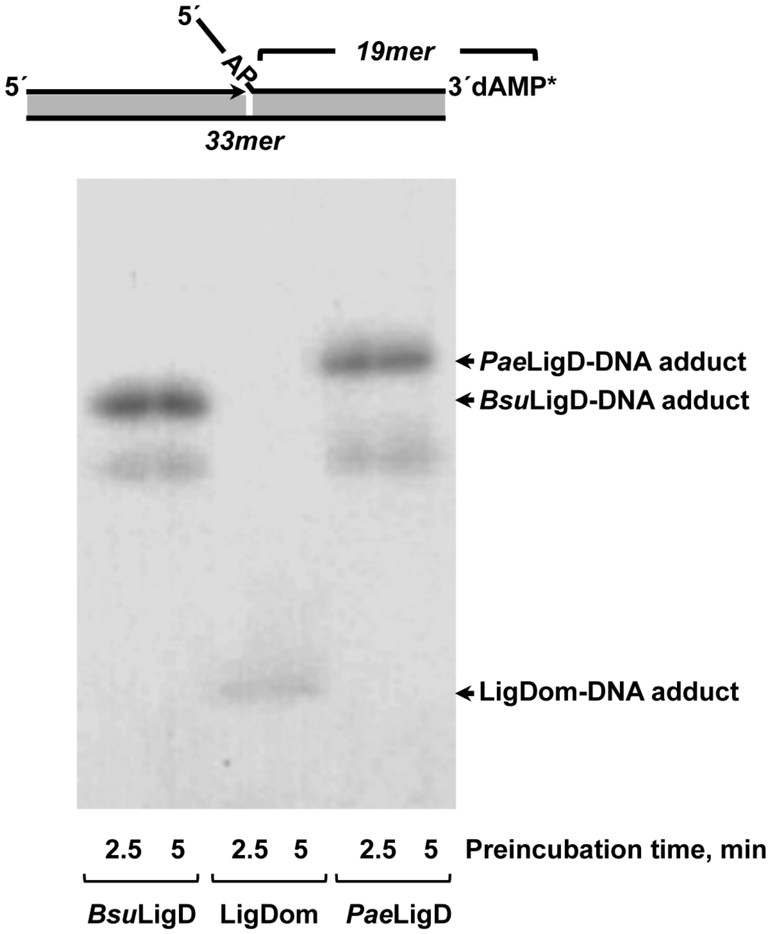

Supplementary Figure S3. Formation of protein-DNA adducts. The assay was performed as described in Materials and Methods by incubating 4 nM of the [^{32}P]3′-labeled uracil-containing oligonucleotide (depicted on top of the figure) pretreated with *E. coli* UDG to leave an intact AP site and further incubated with the indicated protein (100 nM *Bsu*LigD or *Pae*LigD, 147 nM LigDom) for the indicated times in the presence of 0.64 mM $MnCl_2$. After incubation samples were reduced by adding 100 mM $NaBH_4$ and further subjected to SDS–PAGE and autoradiography. The mobility of the different protein-DNA adducts is indicated.

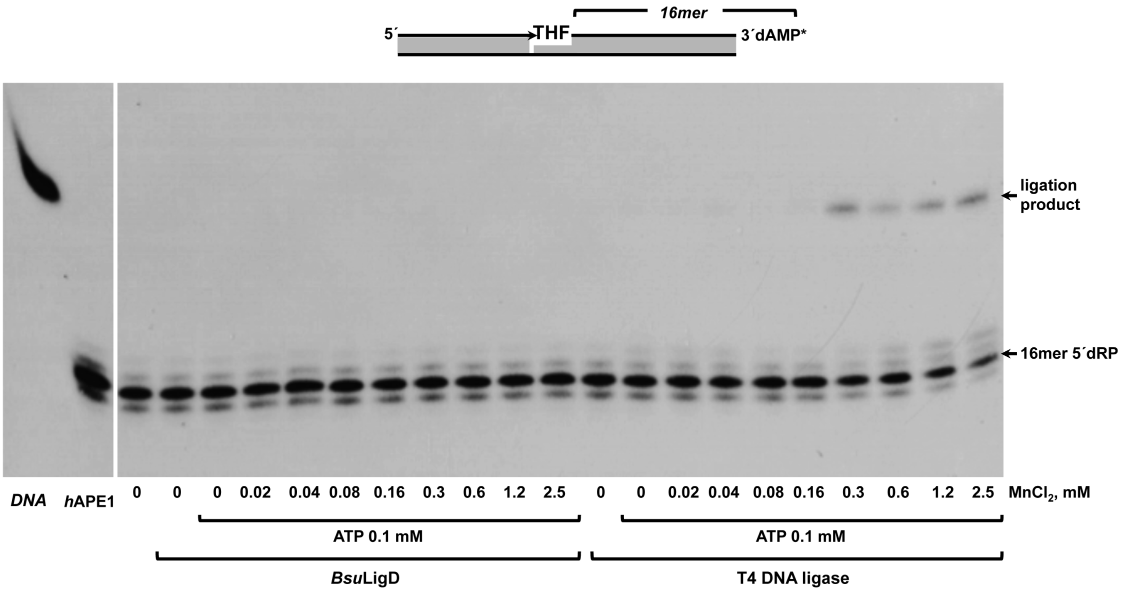

Supplementary Figure S4. *Bsu*LigD does not seal 3´-OH and 5´-dRP ends. A 3´-end labeled (*) oligonucleotide containing a single synthetic AP (THF) site (lane *DNA*) was treated with *h*APE1 to insert a nick at the 5´ side of the molecule (lane *h*APE1). The assay was performed as described in Materials and Methods. Briefly, 0.53 nM of the nicked substrate was incubated with either 57 nM *Bsu*LigD or 40 U of T4 DNA ligase with the indicated $MnCl_2$ concentrations and in the absence or presence of 0.1 mM ATP. After incubation for 30 min at 30ºC, samples were analyzed by 8 M urea-20% PAGE and autoradiography. Position of products is indicated.

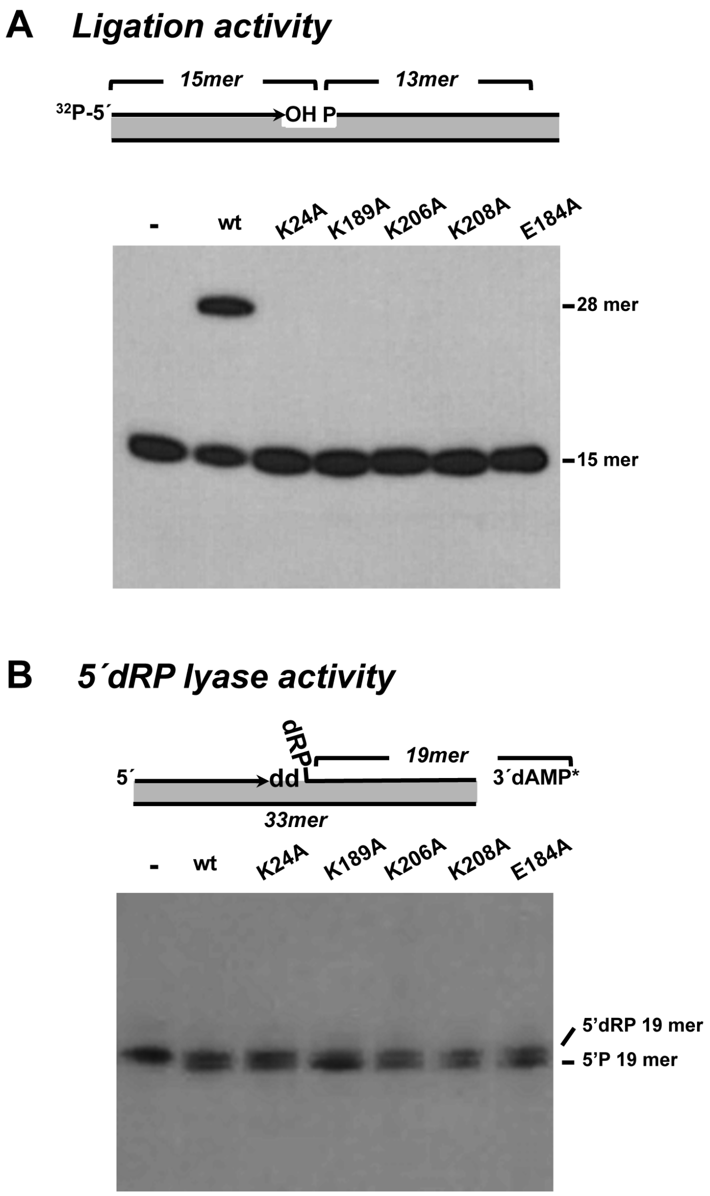

Supplementary Figure S5. Analysis of dRP lyase and ligase activity of *Bsu*LigD mutants K24A, K189A, K206A, K208A and E184A. (A) Ligase activity. The assay was performed by incubating 23 nM of the indicated protein with 1.2 nM of the nicked DNA hybrid F (depicted in top of the panel) in the presence of 5 μM $MnCl_2$. After incubation for 30 min at 30ºC, samples were analyzed by 8 M urea-20% PAGE and autoradiography. (B) dRP lyase activity. The assay was performed as described in Materials and Methods. Briefly, 50 nM of the DNA hybrid B was preincubated with *E. coli* UDG as described above, to render a 5´-dRP end (see scheme of the substrate on top of the panel). Reactions were initiated by adding 28 nM of the indicated protein in the presence of 0.64 mM $MnCl_2$. After incubation for 20 min at 30 ºC, reactions were stopped by addition of freshly prepared $NaBH_4$. Samples were analyzed by 8 M urea-20% PAGE and autoradiography.

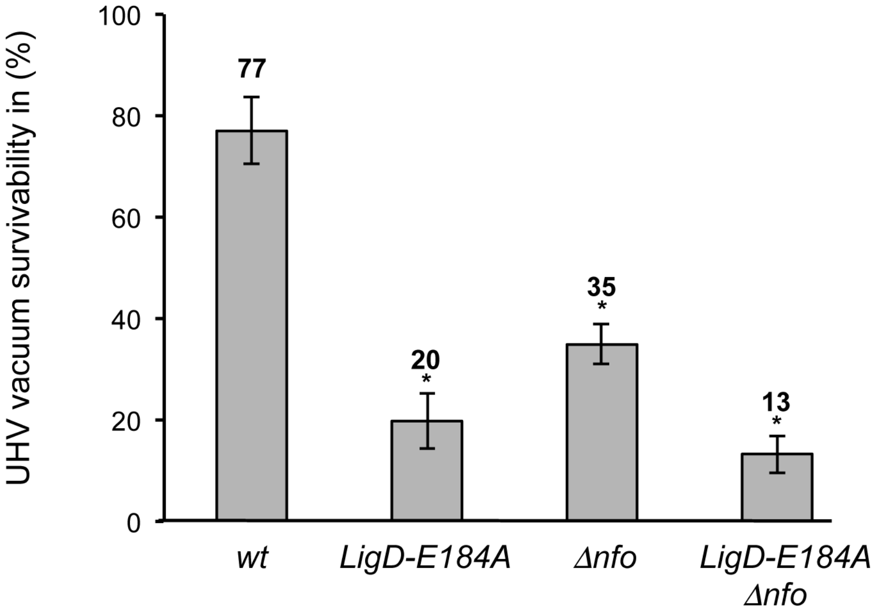

Supplementary Figure S6. Survival of *B. subtilis* spores expressing the *Bsu*LigD ligase deficient mutant E184A and/or Nfo AP endonuclease. The assay was performed as described in Materials and Methods. The CFUs of UHV-treated spores were divided with the average CFU-value of untreated spore samples in order to obtain the survival after UHV. The data presented are expressed as average values±SD, N=4. Asterisks indicate UHV survival values that were significantly different (P-values ≤ 0.05) from values for wild-type (wt) spores.
